# Initial Experience in Monitoring QT Intervals Using a Six-lead Contactless Mobile Electrocardiogram in an Inpatient Setting

**DOI:** 10.19102/icrm.2021.120301

**Published:** 2021-03-15

**Authors:** Daniel R. Frisch, Eitan S. Frankel, Darius J. Farzad, Sang H. Woo, Alan A. Kubey

**Affiliations:** ^1^Division of Cardiology, Thomas Jefferson University Hospital, Philadelphia, PA, USA; ^2^Division of Hospital Medicine, Thomas Jefferson University Hospital, Philadelphia, PA, USA; ^3^Department of Medicine, Mayo Clinic, Rochester, MN, USA

**Keywords:** Contactless care, COVID-19, KardiaMobile, KardiaStation, QT/QTc interval

## Abstract

Mobile electrocardiograms (ECGs) (mECGs) using smartphone applications are an emerging technology. In the coronavirus disease 2019 (COVID-19) era, minimizing patient contact has gained increasing importance. Additionally, increased QT/corrected QT (QTc) monitoring has concurrently been required. The KardiaMobile 6L ECG device, cleared by the United States Food and Drug Administration (FDA) for recording ECGs, along with the KardiaStation tablet application is a platform (AliveCor, Mountain View, CA, USA) that addresses these two issues. A team of residents, fellows, hospitalists, and cardiologists identified inpatients in need of QT/QTc interval monitoring to pilot the adoption of a system composed of a KardiaMobile 6L ECG device with the accompanying KardiaStation tablet application. Concurrent standard ECGs provided validation. Adoption and performance issues were recorded. Four patients agreed to participate in QT/QTc interval monitoring, three of whom were positive for severe acute respiratory syndrome coronavirus 2 viral infection. After basic instructions were given to the patients and their clinical nurses, all patients recorded mECGs successfully. Patients were able to record their own mECG tracings at least once without any assistance. The 12-lead ECGs and mECGs each showed the correct rhythm, and the measured QTc intervals on each modality were consistently acceptable (< 500 ms). Contactless ECGs were successfully uploaded to KardiaStation for QT/QTc interval measurement and archiving. In this study, we showed that an FDA-cleared product, KardiaMobile 6L, has the ability to provide high-quality contactless ECGs for reliable QT/QTc interval measurements. Hospitalized patients were able to perform recordings when requested after receiving simple instructions at the time of first use. This technology has applications during the COVID-19 pandemic and beyond.

## Introduction

Patients with prolonged QT/corrected QT (QTc) intervals are at a greater risk for torsades de pointes and subsequent ventricular fibrillation and sudden cardiac death.^1^ A prolonged QTc interval is a known side effect of several hundred medications, including hydroxychloroquine and azithromycin, two drugs that were used to treat patients with coronavirus disease 2019 (COVID-19) in abundance early on in the clinical experience with this viral condition.^1–3^ Patients with known and suspected arrhythmias are common in outpatient practice.^4^ Advances in mobile technology are widely available and extend the scope of remote monitoring beyond that of conventional medically prescribed products. A commercially available mobile electrocardiogram (mECG) device, KardiaMobile (AliveCor, Mountain View, CA, USA), is relatively inexpensive and works with a smartphone or tablet application; using sensors for fingers from the right and left hands, it provides a 30-second or longer tracing corresponding to lead I of a standard 12-lead ECG. Compared with simple pulse rate monitors, the ability to record ECG information is more useful for arrhythmia diagnosis.^5^ Beyond the ability of patients to record their own mECGs, AliveCor offers a subscription service, KardiaPro, which logs the mECG recordings for collection and review. Since 2017, our institution, Thomas Jefferson University Hospital, has registered outpatients to this encrypted, practice-managed service.

The KardiaMobile mECG device has been used experimentally in a variety of ambulatory settings to detect arrhythmias and conduction system abnormalities such as atrial fibrillation, to monitor QT/QTc intervals during antiarrhythmic drug initiation, and to diagnose myocardial infarction.^6–10^ These proof-of-concept studies largely have depended on single-lead measurements made manually, which provide less information than what is available with a multilead analysis. Previously, we developed a method to record alternate ECG leads (aside from lead I) using the mECG device.^11^ We observed that patients were able to use the technology without difficulty. In May 2019, the United States Food and Drug Administration (FDA) granted clearance to the KardiaMobile 6L device, the first-ever, personal six-lead ECG device; KardiaMobile 6L records six of the 12 leads usually obtained from a standard ECG (ie, the limb leads are recorded but not the precordial leads). A six-lead tracing is recorded by placing a person’s right and left fingers or thumbs on the corresponding electrodes on the top surface of the device while simultaneously placing the electrode on the bottom surface of the device against the skin on the left lower extremity.

On March 23, 2020, the FDA issued an enforcement policy to help expand the availability and capability of noninvasive remote monitoring devices, such as KardiaMobile 6L, to facilitate patient monitoring while reducing patient and health care provider contact and exposure to patients positive for severe acute respiratory syndrome coronavirus 2 (SARS-CoV-2) infection for the duration of the COVID-19 public health emergency. This policy enabled the use of the KardiaMobile 6L device for QT/QTc interval monitoring to improve patient safety while administering potential QT/QTc interval–prolonging medications.^5^

Given the limited experience with using mECGs in the inpatient setting and the use of potential QT/QTc interval–prolonging medications (hydroxychloroquine and azithromycin) in COVID-19 patients, we sought to describe our experience with this technology. The purpose of this case series was to assess the feasibility of recording using the commercially available mECG device, KardiaMobile 6L, along with a tablet application (KardiaStation; AliveCor) in inpatients needing intermittent ECG monitoring. We also sought to document the ease of use of contactless ECG recordings and to compare contactless ECG recordings from the KardiaMobile 6L with standard ECG recordings, which require medical personnel to come into close proximity with COVID-19–positive patients. Our aim was to derive clinical practice lessons for the use of KardiaMobile 6L beyond the COVID-19 pandemic, as we believe that heightened infectious disease protocols minimizing health care provider and patient exposure will be the “new norm” in inpatient management.

## Methods

### Patient population

Eligible participants included male and female COVID-19–positive patients or patients requiring ECG monitoring who were at least 18 years of age. All patients were diagnosed using nasal swabs. Study participants were not on telemetry but required intermittent ECG monitoring and had no prior experience using an mECG device. Among suspected or confirmed COVID-19–positive patients, respiratory isolation precautions were used. Hydroxychloroquine and azithromycin were administered due to the experimental use of these treatments for the management of COVID-19–related clinical sequelae at the time of the study. We included patients with QTc interval values of greater than 500 ms, as we deemed them to be at high risk for torsades de pointes. We excluded patients unwilling or unable to use the KardiaMobile 6L device and those who needed continuous telemetry.

### Endpoints

The primary goals of this study were to assess the feasibility of obtaining recordings using the commercially available KardiaMobile 6L mECG device together with a tablet application (KardiaStation) among inpatients needing intermittent ECG monitoring and to qualitatively compare the results with standard 12-lead ECG recordings. The secondary purpose was to determine whether patients could perform their own recordings with the device when the health care provider is outside the room (ie, a contactless recording). We hypothesized that KardiaMobile 6L would be able to yield an accurate ECG recording in a contactless manner.

### Study procedures

**Figure 1:**
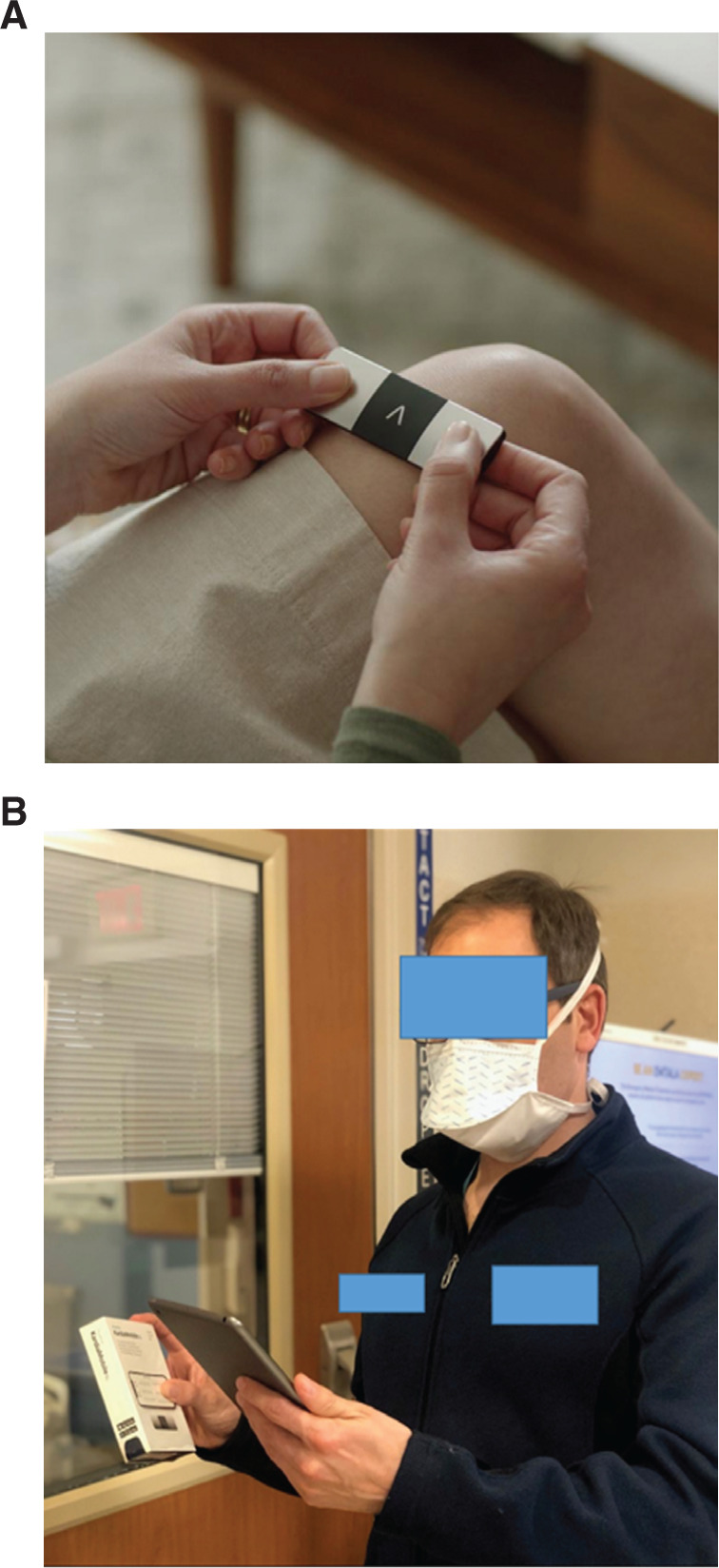
The KardiaMobile 6L device. **A:** By placing two thumbs on the device and holding it against the knee, a six-lead ECG recording can be taken. **B:** A clinician uses the KardiaStation app on an iPad tablet outside of the patient’s room while the patient takes measurements inside their room. Images courtesy of AliveCor.

The manufacturer provided several KardiaMobile 6L devices, a dedicated iPad tablet (Apple, Inc., Cupertino, CA, USA), and a demonstration version of the KardiaStation application **(Figure 1)**. On the same tablet, we created a shortcut to our institutional KardiaPro account. Additionally, we connected to the hospital WiFi and had access to the institutional electronic health records.

The multidisciplinary care team, consisting of cardiologists, hospitalists, and nurses, met initially by video conference to discuss the mECG product and software. Once a hospitalist identified a potential patient, the team members communicated electronically about eligibility for inclusion. A resident or fellow (D. R. F., E. S. F., or D. J. F.) spoke either to the patient’s nurse or directly to the patient to explain the system; consent for patient inclusion in this research was attained during the first encounter between the patient and the cardiologist or cardiology fellow. One of the team members showed the nurse how to complete a recording by using a demonstration device and the iPad. The nurse then had the opportunity to ask any clarifying questions and to ensure she or he understood how to use the device. To minimize direct contact, these conversations were generally held via telephone with the health care provider outside the room. The next time that the nurse went into the room for clinical care, she or he educated the patient on how to use the device. Patients were also provided with written instructions in English. Within their rooms, patients were brought to within 10 feet of the door (Bluetooth range). One of the cardiologists then stood at the closed door while the patient performed the recordings **(Figure 1)**. Patients completed two recordings using the KardiaMobile 6L device with the health care provider outside the room. After a successful recording, the cardiologist let the patient know that the recording had been completed. A nurse was sometimes in the room during the initial recording, but to fulfill other caregiving responsibilities rather than assist with the recording.

Next, WiFi connectivity was verified and QT/QTc interval analysis was requested through the KardiaPro account. Once requested, the QT/QTc interval analysis was performed by BioTelemetry, Inc. (Malvern, PA, USA), an independent third-party QTc measuring service that has performed nearly 4,000 QT interval measurements to date. Within one hour, one of the cardiologists then logged on to the KardiaPro website to review the mECG, the automatic interpretation, and the QT/QTc interval measurements reported by the third-party source. At the time of discharge, the three COVID-19–positive patients kept the provided KardiaMobile 6L devices and were told about the ability to subscribe to the Kardia system as outpatients. At the cardiologist’s or cardiology fellow’s discretion, patients also received nonmobile ECGs for QT/QTc interval monitoring within one day of the mobile recordings.

### Statistical methods

This was a descriptive study without statistical analysis. This study was approved by the Thomas Jefferson University Hospital Institutional Review Board.

## Results

**Figure 2:**
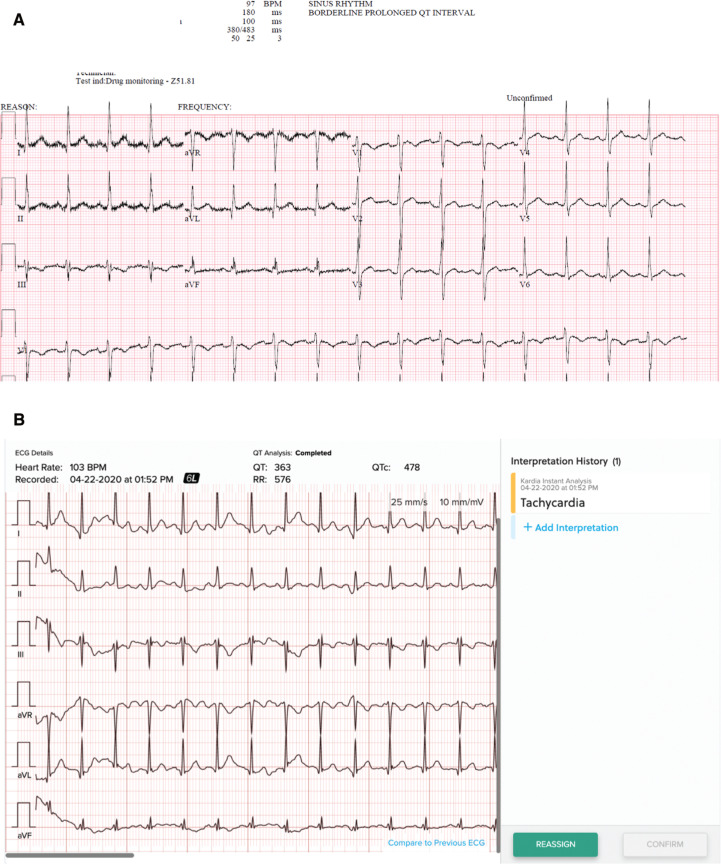

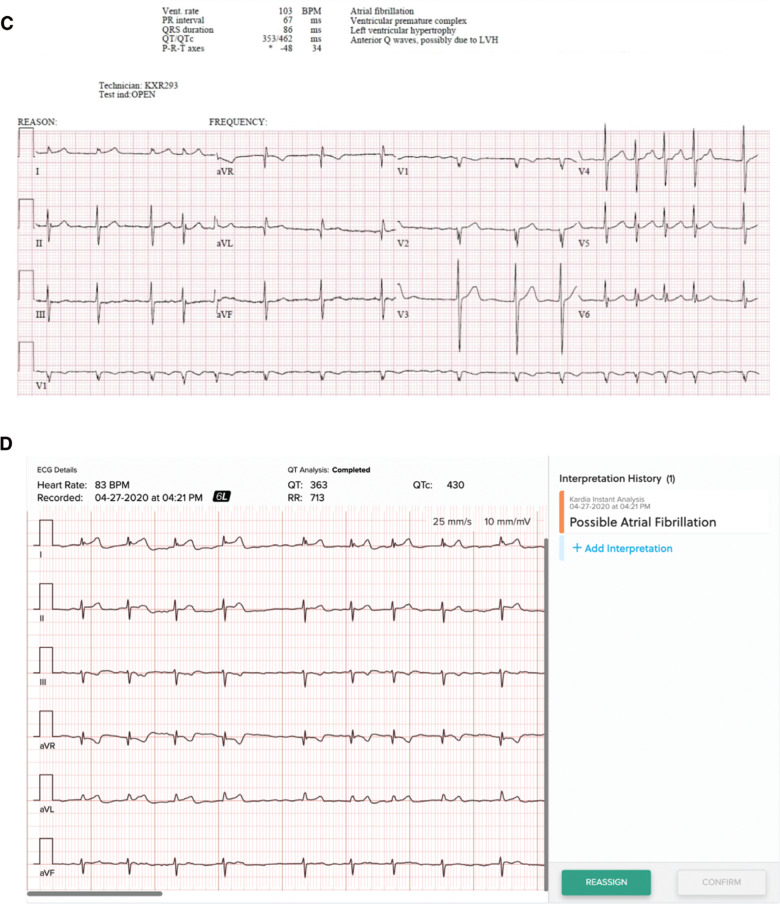
Representative ECGs and mECGs from two patients. **A:** ECG from patient 2. **B:** Six-lead mECG from patient 2. Note that the automatic rhythm interpretations are correct and the QTc intervals are less than 500 ms. Note that 40-ms vertical gridlines have been added to aid in the ability to measure the QT interval manually. **C:** ECG from patient 4. **D:** Six-lead mECG from patient 4. Note that the automatic rhythm interpretations are correct and the QTc intervals are less than 500 ms. Note that 40-ms vertical gridlines have been added to the mECG tracings to aid in the ability to measure the QT interval manually.

Of the six consecutive patients approached for the study, four agreed to participate. The two who did not participate stated that they were not interested. Demographics, ECG results, and mECG results are recorded in **Table 1**. Representative ECG and mECG recordings are shown in **Figure 2**.

### Patient 1

Patient 1 was a 45-year-old man with a medical history of renal cancer (who underwent left nephrectomy and a renal transplant), hypertension, morbid obesity, and sleep apnea. The patient presented with progressive fatigue and intermittent chills for five days and loose stools for one day. On the advice of his nephrologist, the patient presented to the emergency department for evaluation. An evaluation confirmed SARS-CoV-2 infection, and he was started on a five-day course of hydroxychloroquine. He was able to complete two recordings using the KardiaMobile 6L with the health care provider outside the room. At discharge, his inflammatory markers remained elevated but stable.

### Patient 2

Patient 2 was a 48-year-old Spanish-speaking man with no significant medical history who presented with a cough and worsening dyspnea; an evaluation subsequently confirmed SARS-CoV-2 infection. During his hospitalization, he never required supplemental oxygen and was able to ambulate without desaturation while breathing room air. He was given a several-day course of hydroxychloroquine, which was initiated upon admission. He remained on room air with stable inflammatory markers and was discharged with instructions for COVID-19 precautions. His nurse provided instructions to him in Spanish, and he was able to complete two recordings using the KardiaMobile 6L with the health care provider outside the room.

As standard of care, this patient also received nonmobile ECGs for QT/QTc interval monitoring within one day of the mobile recordings. A comparison of the two ECG readings revealed similar automatic rhythm interpretations and QT/QTc interval calculations.

### Patient 3

Patient 3 was a 67-year-old man with advanced systolic heart failure and persistent atrial fibrillation who presented with acute decompensated heart failure. Because of the lack of response to therapies in the intensive care unit, he was transferred to the telemetry ward for consideration of palliative care options. After an unanticipated clinical improvement with diuretic therapy and following further discussion with the patient, he agreed to slowly intensify medical therapy again. However, he requested minimal invasiveness and testing. Given this request, we proceeded with implementing contactless ECG monitoring. Although he did not have COVID-19, his diagnosis of significant cardiomyopathy and anticipated electrolyte shifts clinically justified the use of intermittent ECG monitoring. He was able to complete two recordings using the KardiaMobile 6L device with the health care provider outside of the room.

### Patient 4

Patient 4 was a 96-year-old woman with atrial fibrillation treated with anticoagulation therapy, mild aortic stenosis, dyslipidemia, hypertension, chronic kidney disease, and anemia who presented with subjective palpitations and was found to have atrial fibrillation with a rapid response. One week prior to the current admission, she was hospitalized for near-syncope and was found to be positive for COVID-19. She was treated with hydroxychloroquine for five days and also with ceftriaxone and azithromycin for superimposed pneumonia. Due to variable heart rates, ECG monitoring was required. Despite our initial concerns about this patient’s mobility, “technology literacy,” and frailty, she was able to complete two recordings using the KardiaMobile 6L device with the health care provider outside the room.

This patient also received a nonmobile ECG within one day for validation. A comparison of the two ECG readings revealed similar automatic rhythm interpretations and QTc interval calculations.

### Key lessons

Key lessons from our case series are noted in **Tables 2** and **3**. These include the need for both patient and provider education about the logistics and utility of this ECG method as well as the need for resources beyond the KardiaMobile 6L device itself (ie, a reliable wireless Internet connection and a tablet computer).

## Discussion

mECG devices have previously been used in ambulatory detection of atrial fibrillation with high sensitivity and specificity. In this case series, we found that patients were able to use the mECG product for QT/QTc interval monitoring in an inpatient setting and that accurate recordings were obtainable despite the health care provider not being in contact with the patient. Although not assessed formally, repeat measurements anecdotally required less instruction than the initial measurement and could be completed within minutes. Furthermore, this technology delivered consistent, accurate results among special patient populations (eg, non-English speakers, patients with advanced heart failure, and elderly and frail individuals). Furthermore, we observed that this method of ECG monitoring can be reliably deployed among hospitalized COVID-19–positive patients. Our case series provides evidence that the use of KardiaMobile 6L can likely be extended beyond inpatient COVID-19–related rhythm monitoring and into the outpatient realm.

KardiaMobile 6L uses a unique technology that can record high-quality ECGs of all six limb leads. The technology allows the recordings to be transferred from a mobile device via KardiaStation to an independent diagnostic testing facility that can quantify QT/QTc intervals rapidly and then return the information to frontline health care providers. The recordings from the KardiaStation application automatically synchronize to the KardiaPro physician portal, where clinicians can review the collected data. Unlike most ambulatory ECGs, by virtue of including all six of the limb leads, KardiaMobile 6L includes lead II data, which frequently offer a more accurate view of the QT/QTc interval than data for lead I.^12^

During times of crisis such as the COVID-19 pandemic, when QT/QTc interval–prolonging medications are used, there is high utility in reliably measuring the QT/QTc interval accurately for both inpatient and outpatient use. KardiaMobile 6L offers expedited recordings and, within minutes, can provide information to health care providers. The device is small and recordings can be performed by patients within their rooms. This minimizes close contact with frontline health care providers, potentially reducing the spread of COVID-19 or other infectious diseases to essential personnel and other patients. Furthermore, because of its availability and ease of use, the KardiaMobile 6L device addresses the potential shortage of remote ECG machines in a cost-effective manner given the infection-control protocols.^13^

The use of wearable and patient-owned medical products is likely to increase in the era of heightened infectious disease protocols during and following the COVID-19 pandemic. Minimizing patient-to-provider and patient-to-patient contact is a priority in every hospital process. Essential processes that subject health care providers to high patient exposure, ranging from patient transport to intensive care unit procedures, must be reconceptualized. The process of heart rhythm monitoring is no exception, as placing, changing, and removing ECG leads accurately carry a high risk of exposure for the frontline health care provider. Minimizing this risk illustrates the importance of KardiaMobile 6L and use of an accurate, mobile, patient-centric technology in the inpatient setting. By increasing diagnostic accuracy and by expanding the automated diagnostic possibilities, there is likely to be more widespread acceptance by patients and health care providers regarding the utility of mECG devices and similar technology.

### Limitations

This study was a limited case series of consecutive patients willing to participate. A larger sample may reveal issues that were not observed in this investigation. Furthermore, a larger study would be needed to confirm the accuracy of using patient-operated, externally analyzed mECG devices to detect significant QTc interval prolongation. In addition, due to the limited availability of nonmobile ECGs and telemetry, we were unable to concurrently measure patients using both mECGs and nonmobile ECGs.

## Conclusion

Mobile technology is available for heart rhythm monitoring and has demonstrated diagnostic accuracy and the yield of high-quality ECG recordings. In this study, we showed that an available product, KardiaMobile 6L, had the ability to provide contactless ECGs with acceptable QT/QTc interval measurements. Hospitalized patients were able to perform recordings with simple instructions at the time of first use. Given the heightened importance of minimizing infectious disease risk during and beyond the COVID-19 pandemic, we strongly believe that KardiaMobile 6L can be applied to safely and effectively monitor patients at high risk for arrhythmia in both inpatient and outpatient settings.

## Figures and Tables

**Table 1:** Patient Demographics and ECG Measurements Taken During the Study

Patient No.	Age/Sex/COVID-19 Status	Recording	ECG/mECG Interpretation (Rate)	QT/QTc Interval (ms)	Patient Able to Record ECG Independently?
1	45 years Male Positive	ECG 1	Sinus rhythm (83 bpm)	372/437	N/A
		ECG 2	Sinus rhythm (88 bpm)	372/450	N/A
		Kardia 1	Tachycardia (102 bpm)	360/469*	Yes
		Kardia 2	Normal (97 bpm)	350/445*	Yes
2	48 years Male Positive	ECG 1	Sinus rhythm (97 bpm)	380/483	N/A
		ECG 2	Sinus rhythm (89 bpm)	384/468	N/A
		Kardia 1	Normal (99 bpm)	327/419	Yes
		Kardia 2	Tachycardia (103 bpm)	363/478	Yes
3	67 years Male Negative	ECG 1	AF (97 bpm)	348/442	N/A
		ECG 2	AF (103 bpm)	376/492	N/A
		Kardia 1	Possible AF (98 bpm)	373/462	Yes
		Kardia 2	Possible AF (92 bpm)	417/514	Yes
4	96 years Female Positive	ECG 1	AF (103 bpm)	353/462	N/A
		ECG 2	AF (120 bpm)	339/479	N/A
		Kardia 1	N/A	N/A	Yes
		Kardia 2	Possible AF (83 bpm)	363/430	Yes

AF: atrial fibrillation; COVID-19: coronavirus disease 2019; ECG: electrocardiogram; mECG: mobile electrocardiogram; N/A: not available; QTc: corrected QT.

*Manual measurement.

**Table 2:** Key Lessons Learned During the Adoption of the KardiaStation System

Need to educate the stakeholders, composed of a multidisciplinary team of nurses, cardiologists, other physicians, and patients, about the device and method Information about the product and a demonstration of its use for patients were required
Need the KardiaMobile 6L device and a recording device, such as an iPad, equipped with the KardiaStation application and KardiaPro software to review the recordings and QT interval
Need reliable wireless connectivity to review the tracings and to send the information to other participating services (eg, to BioTelemetry, Inc. for QT/QTc interval measurements)

QTc: corrected QT.

**Table 3:** Key Lessons to Apply Beyond the COVID-19 Pandemic

•	Rhythm monitoring can be accomplished with a simplified WiFi-enabled device and application
•	The ability to record via KardiaMobile 6L expands the tools available to obtain ECG recordings (ie, do not need a 12-lead ECG machine only)
•	Monitoring the QTc interval for values ; 500 ms was reliable in this situation and can be applied during QT monitoring in the context of other drugs (eg, antiarrhythmics)
•	Patients can continue to monitor rhythm, rate, and QTc interval as outpatients with the same device

ECG: electrocardiogram; QTc: corrected QT.
